# What Influences DNA Replication Rate in Budding Yeast?

**DOI:** 10.1371/journal.pone.0010203

**Published:** 2010-04-27

**Authors:** Thomas W. Spiesser, Christian Diener, Matteo Barberis, Edda Klipp

**Affiliations:** 1 Theoretical Biophysics, Institute for Biology, Humboldt University Berlin, Berlin, Germany; 2 Max Planck Institute for Molecular Genetics, Berlin, Germany; Universität Heidelberg, Germany

## Abstract

**Background:**

DNA replication begins at specific locations called replication origins, where helicase and polymerase act in concert to unwind and process the single DNA filaments. The sites of active DNA synthesis are called replication forks. The density of initiation events is low when replication forks travel fast, and is high when forks travel slowly. Despite the potential involvement of epigenetic factors, transcriptional regulation and nucleotide availability, the causes of differences in replication times during DNA synthesis have not been established satisfactorily, yet.

**Methodology/Principal Findings:**

Here, we aimed at quantifying to which extent sequence properties contribute to the DNA replication time in budding yeast. We interpreted the movement of the replication machinery along the DNA template as a directed random walk, decomposing influences on DNA replication time into sequence-specific and sequence-independent components. We found that for a large part of the genome the elongation time can be well described by a global average replication rate, thus by a single parameter. However, we also showed that there are regions within the genomic landscape of budding yeast with highly specific replication rates, which cannot be explained by global properties of the replication machinery.

**Conclusion/Significance:**

Computational models of DNA replication in budding yeast that can predict replication dynamics have rarely been developed yet. We show here that even beyond the level of initiation there are effects governing the replication time that can not be explained by the movement of the polymerase along the DNA template alone. This allows us to characterize genomic regions with significantly altered elongation characteristics, independent of initiation times or sequence composition.

## Introduction

One of the basic traits of all living systems is the ability to reproduce and transmit genomic information to their offspring. The requisite for that is exact and efficient replication of the genome, a highly controlled cellular process, which makes up a large part of the cell cycle. Severe malfunctions within DNA replication are usually lethal. As such, DNA replication is subject to a complex regulation in all eukaryotic organisms, which makes the identification of the underlying mechanism a non-trivial task.

In a cell, DNA replication begins at specific locations scattered across the genome called origins of replication [1]–[3]. They contain DNA sequences that are recognized by replication initiator proteins, such as the dnaA in *Escherichia coli* or the origin recognition complex (ORC) in yeast, which in turn recruit other proteins to separate the two strands and initiate replication [4]–[6].

A central role in the process of replication is played by activation of helicases, which break the hydrogen bonds holding the two DNA strands together and generate two single strands of DNA. In the budding yeast *Saccharomyces cerevisiae*, the ORC complex bound to the origin initiates Mcm2-7 helicase loading in G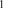 phase by recruiting specific licensing factors in the pre-replicative complex (pre-RC) [7]. When cells enter S phase, the activation of kinase complexes - Cdk1-Clb5,6 (S-CDK) and Cdc7-Dbf4 (DDK) [8], [9] - regulates the Mcm2-7 helicase [10], [11]. Once activated, Mcm2-7 unwinds origin DNA to trigger the initiation of DNA replication [12].

This unwinding of DNA at the origin and synthesis of new strands form a replication fork at which the replication takes places in a non-symmetric manner. In the 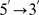 direction, the new DNA strand, also called the leading strand, is synthesized in a continuous manner by the DNA polymerase 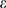 [13]. In contrast, the DNA strand at the opposite side of the replication fork, the lagging strand, is formed in the 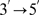 direction. Because DNA polymerase 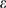 cannot synthesize in this direction, DNA along the lagging strand is synthesized in short segments known as Okazaki fragments [14], [15]. In this process, the DNA polymerase 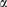-primase complex builds RNA primers in short bursts along the lagging strand, enabling the DNA polymerase 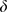 to synthesize DNA starting from these primers in the 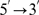 direction [13]. Afterwards, the RNA fragments are removed and the DNA ligase joins the deoxyribonucleotides together, completing the synthesis of the lagging strand (see [16], [17] for recent reviews).

In general, two replication forks emerge from an activated origin of replication, traveling in opposite directions. The rate at which the DNA is replicated can differ between replication forks issued from the same origin, as well as for those from the other origins located on the chromosome. This results in a broad distribution of replication fork rates in budding yeast [18]. Different fork rates at different chromosome regions could have either regulatory functions or could be caused by higher order structures of the chromosome (e.g. protein binding or tertiary structure). However, what exactly causes deviations in the replication fork rates has not yet been established satisfactorily. It has been suggested that epigenetic alterations influence fork rates both in yeast [19]–[24] and in higher eukaryotes [25], [26], and that chromatin structure could modulate origin activity [27]. Furthermore, transcriptional activity regulates the replication origin activity [28]–[30] and it seems possible that it might play a role in altering the replication fork progression [31]–[33], even though it is not clear whether it enhances the fork rate due to already partly unwound DNA or impeding it because the DNA is blocked by proteins involved in transcription.

Besides epigenetic factors, also availability and abundance of single nucleotides affect activation of origins of replication [34] and could play a role in variations of replication fork rates. Fork rates are generally established by a directed movement of the replication machinery along the DNA template. The polymerase has to advance nucleotide per nucleotide, apparently making the process itself non-continuous, with a stepwise character. This is due to the movement of the complex from a replicated nucleotide to the next unreplicated one (movement step), that is interrupted by the catalyzing activity during which the complex is stationary on the DNA. During the stationary state, the replication machinery incorporates a nucleotide into the nascent DNA strand that corresponds to the one of the template. This process is subject to various fluctuations, like nucleotide-specific polymerization kinetics, substrate availability (diffusion of the nucleotides), mismatch control (wrong nucleotides arriving at the polymerization sites but not being processed) and malfunctions that potentially cause delays. This makes DNA replication motion at least partly a stochastic process that is dependent on sequence properties such as length and base composition. However, to which extend this contributes to the overall replication rate remains unclear, and whether these sequence-specific attributes play an active role in the variation of DNA replication rates has, to our knowledge, not been investigated.

In this work, we interpret the replication machinery movement as a directed random walk. A directed random walk can be seen as a process in which the location of an object randomly changes by a single directed step, depending on some probability parameters. In the case of the replication machinery, the directed step is the movement with probability 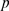 or the stalling/waiting with probability 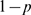. The replication machinery only moves if the appropriate nucleotide is instantly available and can be incorporated without problems, and stalls in case of a mismatch or other fluctuations, as mentioned above. The movement of the machinery takes the characteristic time 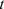 and the stalling takes the time 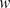. Probabilities (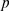), transition times (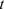) and waiting times (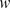) may be specific for the four bases A,T,G and C.

A general assumption of this work is that observed replication rates, that can be found in literature, are governed by two different and independent aspects, one that is sequence-specific and one that is not. It is the combination of both aspects that probably determines the shape of the experimental replication profiles [18] and the dynamics of DNA replication. However, it is currently not known to which extent both factors contribute to the observed dynamics, nor whether these contributions are locally restricted or not. There are global properties influencing the replication rate (like the nucleotide composition), as well as e.g. histone acetylation/methylation or active transcription, which vary throughout the genome and are therefore rather local quantities. We assume that the replication time of the profiles (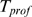) is composed of the following: the time that the replication machinery needs in terms of reaction kinetics (nucleotide incorporation) and motion (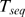), the time that is needed to account for active transcription or any other local regulation (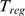) and an error (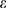) standing for random fluctuations, thus: 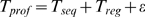. This equation also exemplifies our approach: we decompose the experimental data (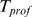) into the different components. We do this by describing and therefore capturing the underlying, seizable part of the system (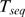) filtering it from the data, to unravel the error (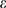) and the unknown, regulatory component (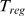) of the data. We provide here a concise characterization of sequence-specific replication rates, as well as a spatial map of regions with sequence-independent alterations in replication rates within the genomic landscape of budding yeast.

## Methods

### Model formulation and assumptions

**Figure 1:**
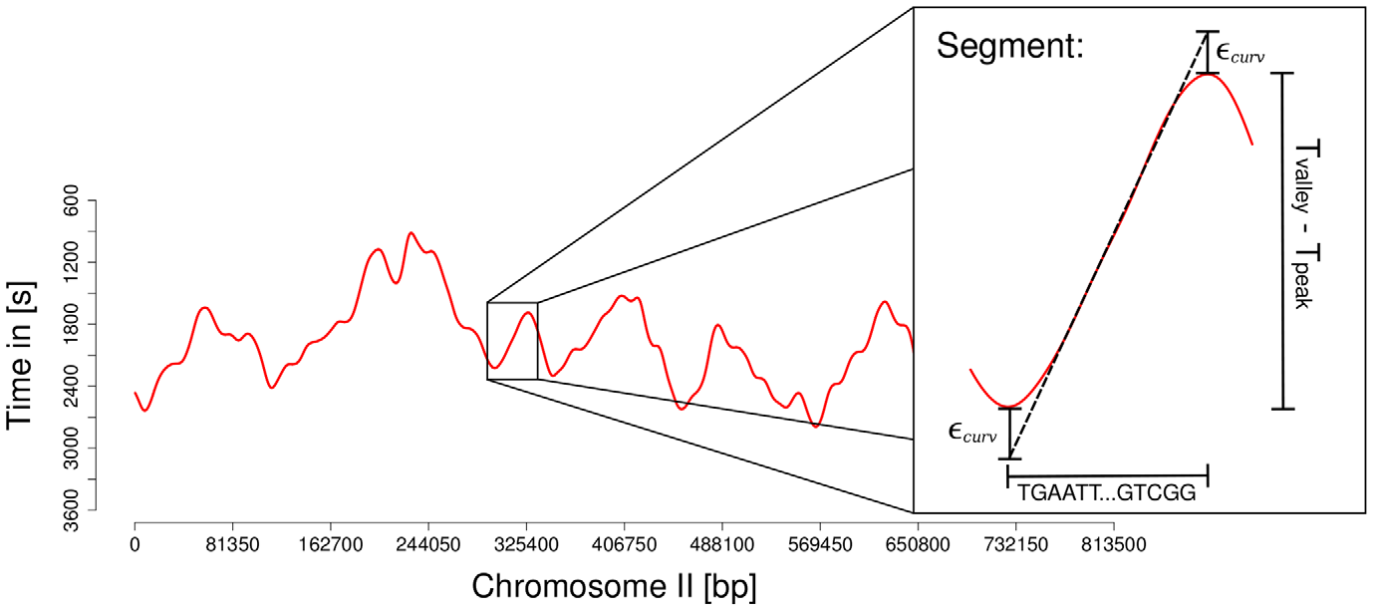
Schematic view of the data processing procedure. The genomic sequence between one peak and one valley in the experimental profiles (Chromosome II is shown as an example [18]) is called “segment”. We calculated the elongation time as the time difference between the corresponding peak and valley, where 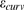 denotes the error caused by data smoothing.

Genomic sequences for all the 16 chromosomes of budding yeast were obtained from the NCBI reference sequences database [35]. Information about the replication dynamics in budding yeast was extracted from recently published whole genome replication profiles [18]. A replication profile is the plot of the replication time as a function of the position in the chromosome (as an example, the profile for chromosome II is shown in Fig. 1). Peaks correspond to replication origins and valleys to termination zones. The earlier an origin initiates DNA replication, the higher is its respective peak in the profile. The initiation process is also called origin firing. The slope of the line connecting an origin (peak) and a termination zone (valley) shows the direction and the rate of the fork migration. Replication profiles represent an average of population and not single cell data, therefore, caution must be taken in directly relating those profiles to the elongation time of the individual replication forks. The authors calculated the profiles as means over several individual measurements, therefore we can not expect to characterize the level of variation within the data and, thus, the inherent stochasticity. However, it is possible to calculate the mean value of the stochastic process that governs the replication dynamics. Additionally, profiles obtained from the literature have been smoothened prior to publication and thus been transformed to a continuous curve where the original peaks and valleys of the profile at the replication origins are flattened. This leads to a slight distortion of the data. We approximated the maximum error this effect imposes on the replication profiles. This error can be quantified by measuring the lengths of chromosomal regions within the profile that show a non-zero curvature, thus 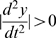. Multiplying the lengths of those regions, 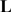 (in base pairs), by the inverse of the average overall replication rate, 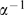 (in seconds per base pairs), yields the error distribution
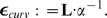
(1)Furthermore, the profiles contain the combined information of the initiation (or firing) time of the origins and the time required for the elongation for every chromosomal regions. In this paper we shall refer to the genomic sequence between one peak and one valley in the profile as a “segment”. For those segments we calculated the elongation time as the time difference between the corresponding peak and valley (as shown in Fig. 1). Thus, a single segment 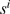 is assigned to a single elongation time 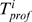 which we decomposed into 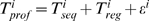. For 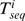 we allowed a direct dependence on the nucleotide composition of the sequence, which is the frequency of each nucleotide within the segment. The remainder consists of a normal-distributed error term 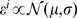 and a specific time 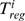. 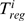 denotes some unknown local influence on the replication time and is not following the normal distribution of the error. We allowed a non-zero mean (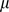) here since we might have systematic global errors. For example 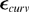 is also contained in 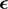. This directly imposed a statistical test for identifying segments with a non-zero 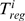 by comparing against the null-hypothesis of the error distribution of the 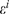. For this aim, we filtered the individual 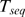 from the elongation times 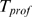 by building a mathematical model which specifically describes 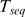.

Here, we assumed that the replication machinery movement on the DNA segment follows a directed random walk where the probabilities for the movement and the corresponding waiting and step times were only dependent on the current position (base) of the replication machinery and independent of the previous or next position. Furthermore, since the data of Raghuraman et al. only indicate the movement of the replication machinery and does not give detailed information about leading and lagging strand polymerization, we made further assumptions. The following components are not modeled explicitly but assumed as part of the replication machinery: helicase Mcm2-7 with associated factors, polymerases 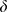 and 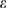, polymerase 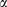-primase and ligase. We further assume that the synthesis of the leading and the lagging strand occurs in parallel.

For the movement we assumed that the replication machinery would either move forward with a base-dependent probability 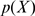 for base 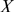 or wait with probability 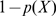 (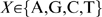). For a finite sequence this yields a total step number 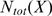 for each base being the sum of forward (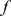) and waiting (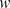) steps (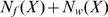). Here the forward step would take a characteristic time 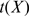 and the waiting step a time 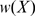 (illustrated in Fig. S1). Due to the spatial independence the probability for 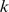 forward steps for base 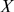 now follows a binomial distribution, thus
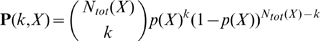
(2)with expected forward steps
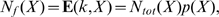
(3)where 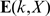 denotes the expectation of the binomial distribution. However, since 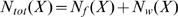 and 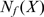 being the (expected) number of forward steps for base 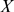, we can derive the expected number of waiting steps by the number of forward steps, since
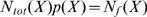
(4)

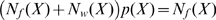
(5)

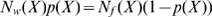
(6)

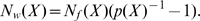
(7)


This formulation is important since the information we get from the profiles is the number of forward steps for each of the bases (simply the base counts in the segment). Thus, receiving the number of forward steps for each base 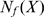 from the segment lengths we can now derive the expected replication time as the sum of times required for each subset of bases,
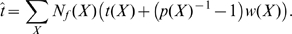
(8)


Defining the column vectors 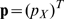, 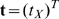 and 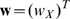 and setting 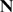 to be the 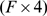 matrix with the base counts for each of the F segments in its columns, we can concisely derive the segment-depending replication times via
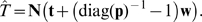
(9)Equation (9) is, under the given assumptions, the most general description of the time required for the replication of a single segment. We call it here model 1. It is the most complex model because it allows different parameters for each of the four bases (12 parameters in total).

However, one may also drop some of the assumptions in order to reduce the complexity of the model and test whether the four bases have the same influence. In this special case, where we assume independence of the base itself, the matrix 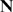 becomes a column vector where each row entry denotes the length of the segment and the parameter vectors become scalar. The approximated replication times are then given by
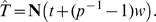
(10)The description in equation (10) is called model 2. It uses the same parameters for each of the four bases (3 parameters in total).

Finally, we further simplified the model to a version where the second term was summarized into a single parameter 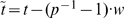, yielding a completely linear model of the form
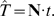
(11)Equation (11) is the most simple description, called model 3: an average replication time per base multiplied with the length of the segment.

All filtering has been done with the most detailed description we derived (model 1). The other two models were solely used for model comparison.

### Model fitting

The models 1 and 2 were fitted to the experimental data [18] by an initial global regression step followed by a local refinement step. The global step was performed using Simulated Annealing with a modified sampling step, where we used a kernel of truncated normal distributions in order to include boundaries for the parameters (all parameters were assumed to lie within [1e-8, 1]) [36]. The local refinement step was executed using the L-BFGS-B algorithm with the same boundaries [37]. As a goal function we chose the sum of squared residuals given by the measured values 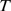 and the approximated values 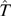, given by 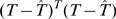.

The regression was performed for 1000 uniformly distributed initial values (in the range [1e-8, 1]) for the parameters which enabled us to derive the parameter correlations. The remaining replication times, or filtered times, were then calculated as the difference of the experimental and the mean of the fitted replication times (
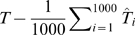
) and their distribution and remaining correlation to the segment lengths was computed. For all correlation measures, we used the Spearman rank correlation [38]. In order to quantify the effects independent of the underlying sequence or segment length the filtered times were first approximated by a normal distribution. The rationale behind is that a normal distribution would indicate a combination of random processes being responsible for the residuals whereas all deviations from that distribution would indicate some form of regulation. The parameters for the normal distribution were approximated by robust measures, namely the median for the mean and the median absolute deviation (MAD) for the standard deviation. In a second step we identified all segments whose remaining replication time (deviation from the approximated segment-dependent replication time) was significantly different from the prior normal distribution on a significance level of 0.05 with the Holm-Bonferroni correction applied [39]. This also ensured that the smallest significant remaining replication time was still larger than the largest error which we can expect due to the smoothening of the profiles. Thus, the significance can not be explained by the data smoothening.

### Model ranking

In a last step we ranked the models according to the Akaike Information Criterion (AIC) [40]. The AIC is a tool for model selection, which means it can be used to compare competing models with one another. It quantifies the information that is lost when an estimated statistical model is used to describe reality and combines this goodness of fit with the complexity (degrees of freedom) of the model. The model with the lowest AIC value of the model ensemble is the best. The AIC value is a relative measure and therefore not suitable for single model evaluation but only ranking within a model ensemble. Here, the AIC has been calculated on the basis of two different statistical measures, the residual sum of squares (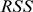) and the coefficient of determination (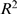) as follows
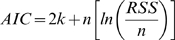
(12)with 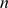 equal to the number of observations and 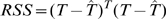. Furthermore,
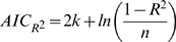
(13)with 
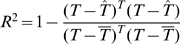
 where 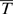 is equal to the mean of 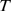.

All tasks were implemented and analyzed with the R statistics environment [41].

## Results

### Elongation times are directly related to the segment lengths for a large part of the genome

On the assumption that the observed replication profiles can be decomposed into a sequence-related part and a non-related part (see introduction), we built a stochastic model for the replication machinery movement to characterize the first part of the equation 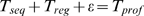. Therefore, the model must be able to capture the two different attributes of 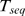 that matter the most: differences in base composition of the DNA and differences in lengths of segments.

**Figure 2:**
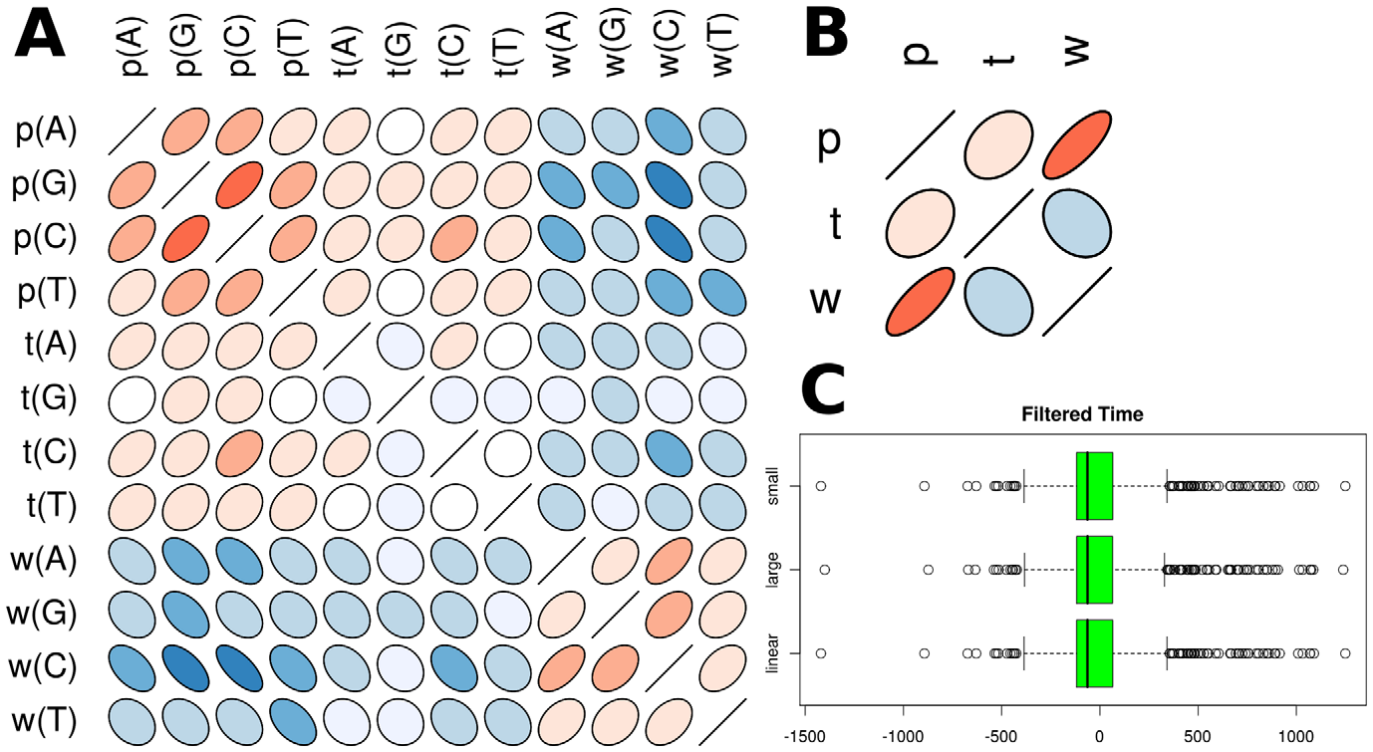
Model comparison. [A] Correlation matrix for model 1. The shape of the ellipses correspond to 95% confidence regions of a Gaussian kernel with the given correlation, as such the longer diameter of the ellipses specifies the direction of correlation whereas the smaller diameter describes how the data deviates from the line of correlation. Orange and blue colors indicate positive and negative correlations, respectively. [B] Correlation matrix for model 2. [C] Filtered times for the three models.

We found a large dependency between the segment lengths and the experimental replication times (correlation coefficient 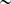
0.82 (Supplementary Information, Fig. S2)). On the contrary, we found almost no dependency between the replication times and the base composition of the segments. The correlation matrix for the 12 parameters of model 1, calculated as described in Methods, shows that they are correlated in a block-like manner (Fig. 2A). The blocks represent probabilities for the movement of the replication machinery, the transition times and the waiting times. All probabilities for the single nucleotides are slightly positively correlated to the transition times (white and light orange ovals) and negatively correlated to the respective waiting times (light blue and blue ovals). A small negative correlation between transition and waiting times (violet and light blue ovals) is observed, however, the intensity of the correlations differs amongst them. Nevertheless, we notice that the higher the chance that the replication machinery moves across a certain nucleotide, the shorter are waiting times in case the polymerase stalls (Supplementary Information, Figs. S3 and S4).

Fig. 2B shows a similar, yet inversed, trend for the 3 parameters of the small model. The transition probability is highly positively correlated to the waiting time (orange oval). The transition time is, if so at all, slightly positively correlated to the transition probability (light orange oval) and slightly negatively correlated to the waiting time (violet oval). In other words, the higher the chance that the polymerase moves at all, the longer it waits in case of stalling.

Fig. 2C shows the filtered times for the three models. Even though the models differ in the number of parameters, model 1 cannot describe the experimental data more accurately than the smaller model 2 or even the linear model 3. Despite the difference in degrees of freedom, the residual sum of squares is only slightly smaller (0.05%) for model 1 compared to the small and the linear ones (Table 1).

**Table 1:** Model statistics and ranking.

	Large	Small	Linear
RSS	42682347	42701178	42701178
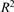	0.535819751983114	0.535614953567048	0.535614953574985
AIC	7425.48716598	7407.78070659	7403.78070659
AIC 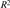	16.7267336032	−1.27282528956	−5.27282528958
Rank	3	2	1

Residual Sum of Square (RSS), Coefficient of determination (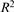), general Akaike Information Criterion (AIC), Akaike Information Criterion based upon the Coefficient of determination (AIC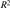) and the model rank are shown.

Model ranking yields that relative to the different number of parameters the linear model 3 performs best, the small model 2 second best and model 1 worst. The detailed model does not fit the experimental data significantly more accurate than the smaller or the linear model. This indicates that the effect which determines the velocity of the replication machinery is largely independent of the composition of the sequence that is to be replicated. If there are differences in transition probabilities, transition times or waiting times between the nucleotides, their contribution is too small to finally determine replication rate deviations. This also holds for nucleotide pairs and triplets (data not shown). Thus, apparent deviations in the replication rate cannot be explained by differences in the sequence composition. Furthermore, despite the huge amount of experimental data points, model 1 as well as model 2 seem to be over-determined; too many parameters show correlation, which indicates that one parameter can be enough to characterize the replication rate in budding yeast, as we recently proposed [42].

**Figure 3:**
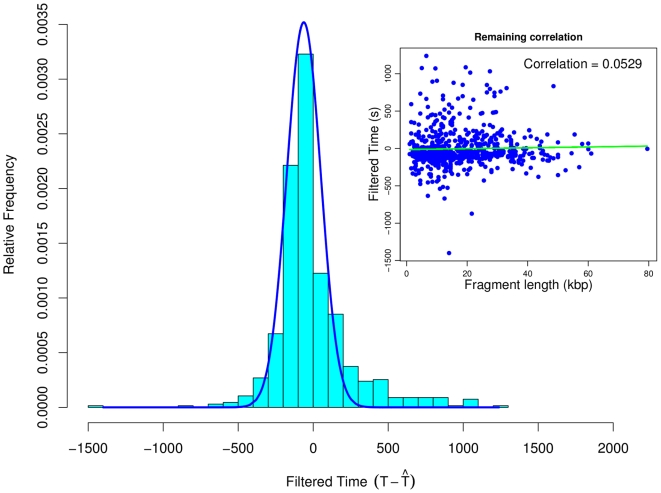
Histogram of the filtered times. The filtered times are calculated as experimentally measured replication times minus the mean of the approximated replication times. They are compared against a normal distribution with mean = −62.7735 and standard deviation = 113.3735, which is shown as well.

Since base composition does not seem to play a major role, and in order to test how much of the length specific correlation is captured by the model, we calculated the correlation coefficient for the filtered times and the segment lengths (Fig. 3). This value was significantly smaller (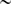
0.05), which indicates that there is hardly any correlation left between the length of the replicated segment and the rate at which it is replicated. In conclusion, we succesfully filtered out 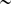
95% of all sequence-specific rate deviations (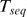) from the experimental data (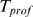).

### Regions with strongly altered elongation distinctly map onto the budding yeast genome

**Figure 4:**
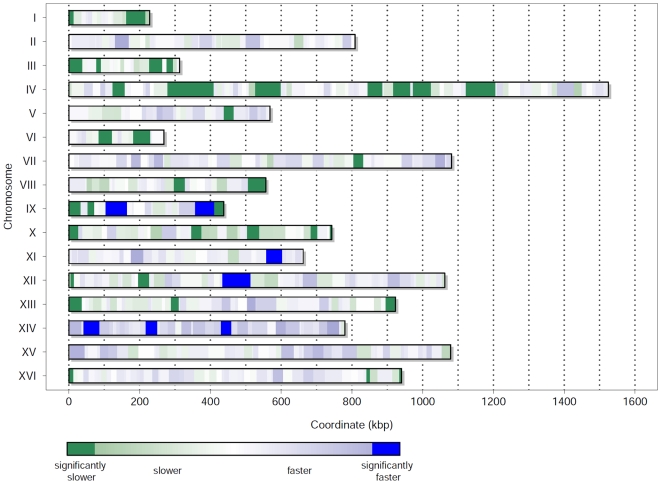
Regions of replication speed deviation for the 16 yeast chromosomes. Deviations within the filtered times across the genome of budding yeast are shown. Blue shades indicate faster replication in the experiments than predicted by model 1, whereas green shades indicate slower replication in the experiments (linear scale, lighter tones indicate smaller deviations). Dark shades indicate a significant deviation from the prior normal distribution. A quantitative view of the deviations (in seconds) for each chromosome can be found in the Supplementary Information (Fig. S5).

The remaining component of the data is now 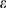 and 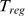, which can be observed in Fig. 3. We found that our model (model 1, average of 1000 different parameter sets), indicated by the median of the filtered time histogram, is slightly too slow (Median = 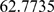 seconds). However, on a time scale of up to 1500 seconds, this is an error of only 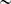
4%. Furthermore, we observe a lower and an upper tail of the filtered time distribution, which are prominently placed outside the overlying normal distribution. These tails indicate DNA segments where the model predicts much faster or slower replication than observed in the experiments. The upper tail is more prominent compared to the lower one. However, it seems that, since the times are already filtered, in both regions other mechanisms, different from segment composition or length, influence the rate of DNA replication. We visualized all regions of replication rate deviation for the 16 chromosomes of budding yeast (Fig. 4). The chromosomal regions that replicate faster in the experimental data compared to the predictions of model 1 are shown in blue, whereas the regions that replicate slower are shown in green. The magnitude of the deviation is indicated by the intensity of the colors.

We found that only few regions replicate faster (blue), whereas many regions show significant delays in DNA replication (green). In particular, we found that only two regions on chromosome IX, one region on chromosomes XI and XII, respectively, and three regions on chromosome XIV replicate significantly faster. On the opposite, the regions where replication is delayed are more frequent and scattered over nearly all chromosomes (except for chromosomes II, XIV and XV). No significant deviations could be detected only for chromosomes II and XV. The exact landscape of the filtered times and the original profiles from Raghuraman et. al. for all 16 chromosomes can be found in the Supplementary Information (Fig. S5). We did not observe that regions with strongly altered elongation correlate with late or early firing origins.

Altogether, our results indicate that replication times in DNA replication are due to, and therefore can be split into, a sequence-specific and a sequence-independent component. Within the sequence-specific part, it is rather the segment length than the segment composition that has an influence on the replication time, which is why the linear model fits almost as good as model 1. It seems intuitive that the larger the segment of DNA is, the longer the replication time. Nevertheless, filtering this fact from the data enabled us to physically locate and map sequence-independent components with a certainty of 95% under the prior normal distribution. From looking at the map, it becomes apparent, that rate deviations that are caused independently of the underlying sequence, are not scattered randomly across the genome, but are clustered on distinct locations within the genomic landscape of budding yeast.

## Discussion

In this work we aimed at quantifying effects that influence DNA replication time in budding yeast. We described the movement of the replication machinery along the DNA template as a directed random walk. By using this approach, we decomposed influences on DNA replication time into two major components, a sequence-specific one and a sequence-independent one.

We have shown that the nucleotide composition of a segment does not significantly influence its replication time. Obviously, we cannot rule out completely that there is a nucleotide composition-specific effect on the replication time. It seems intuitive to assume that there are fluctuations, e.g. in the availability of nucleotides in the nucleus. In our analysis, the probabilities 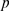 can be viewed as an expression of such fluctuations. They summarize a mixture of factors, incorporating the nucleotide availability among others. However, the contribution of nucloetide composition seems to be too small to be detected by our method using the experimental data taken from Raghuraman et al. [18].

We have demonstrated a strong correlation between segment length and replication time. Once again, this seems to be intuitive, since we can assume that the longer a segment is, the more time it will take to be replicated. Nonetheless, we filtered these two results (non-nucleotide-dependency and length correlation) from the replication times. What we were left with was a distribution of replication times, independent of sequence and length. From the filtered replication times we could directly infer the distribution of replication rates, since all length-specificity is filtered out. This means that, if the replication time is longer than average, the rate would be decelerated and vice versa. The distribution of filtered times was then approximated by a normal distribution. We assumed that all deviations from that normal distribution indicated some form of regulation. Applying this logic, we physically located and mapped sequence-independent components with a certainty of 95%. We observed that regions with significant deviations (violating the assumption of normal distribution) do not show uniform spatial distribution but are clustered on distinct locations, which forms a regulatory landscape within the budding yeast genome. Thus, a large part of the elongation time is dictated by some spatial and sequence-independent factors. We therefore present evidence for another aspect, beyond initiation and origin timing, of the puzzle that is the understanding of regulation of DNA replication in time and space.

However, what exactly regulates DNA replication in the regions where we observed a significant faster or slower replication (see Fig. 4) is not clear. Although, it has been shown that epigenetic factors can influence DNA replication, none of them directly corresponded to the regions we identified [19]–[24]. Nevertheless, an inhomogeneous histone acetylation/methylation pattern could lead to differences in DNA unwinding efficiency, which might cause the observed effect. Histone modification status and remodeling of the chromatin structure could influence the rate at which the replication machinery operates. In fact, particular dense packing of the DNA tertiary structure could account for deceleration of the replication rate and, therefore, modulate origin activity as well [27]. On the other hand, loosely packed or already unwound DNA, due to e.g. transcription, could facilitate replication [31]–[33]. However, it is still under investigation whether these mechanisms of regulation are tightly related to DNA replication or if they are merely the side effects of the regulation of other processes, e.g. transcription. At this point, the reason for the observed local deviations in the replication times remain unclear, but this might be changed as more and more experimental data become available. There is a number of experiments that could be directly infered from our results, e.g. transfer a significantly slower or faster replicating segment to another location in the genome and check whether the replication time is conserved, or mutate the sequence of this segment to investigate the potential changes of the elongation time. Considering the tight connection between DNA replication and the other cell cycle events, a link between the replication speed and the accessibility of the origins is likely. In particular, this is presumably the case for origins that show delayed replication due to the chromatin state of the chromosomes [27] or to the Cdk1-Clb5 activity [43], [44].

On a different note, in this work we have shown, by using the Akaike information criterion [40], that the replication rate in budding yeast can be best approximated using only a single parameter, as we have recently proposed [42]. Naturally, one could argue that we did only test models that consider sequence-specific attributes and no spatial regulatory events. However, we have shown that spatial regions of interest are not randomly distributed, which is why they can only be described explicitly.

In a further development of the analysis presented, we anticipate to relax some of our modelling assumptions. For example, in budding yeast, polymerases 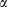, 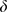, and 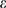 are localized to early firing origin regions during early S phase, suggesting that they function together at multiple replication forks [45]. Their contribution for the apparent speed of the DNA replication process however, has still to be highlighted. In this direction, our study could be suitable for further investigation of their distinctive roles and velocities in the polymerization process. As soon as more experimental data regarding the polymerase kinetics will become available, our model could be extended. In addition, it could be interesting to further investigate stochastic components of DNA replication dynamics in budding yeast. Since S phase dynamics depends both on the replication fork velocity and the initiation frequency of origins, an interesting aspect is to combine time-dependent changes in the replication origin activation and a fork density-dependent affinity of the different polymerases for the origins.

## Supporting Information

Figure S1 Schematic view of the DNA replication model. The replication machinery can move forward with a base-dependent probability *p*(*X*) for base *X*, taking a mean time *t*(*X*) for the forward step and a mean time *w*(*X*) for the waiting step.
(0.03 MB PDF)

Figure S2 Estimated parameters for model 1. Histograms for the 12 parameters as obtained from 1000 independent optimization runs with uniformly distributed initial values. CV denotes coefficient of variation (standard deviation/mean).
(0.04 MB PDF)

Figure S3 Estimated parameters for model 2. Histograms for the 3 parameters as obtained from 1000 independent optimization runs with uniformly distributed initial values. CV denotes coefficient of variation (standard deviation/mean).
(0.02 MB PDF)

Figure S4 Dependence of replication times on the lengths of the DNA templates. In the experimental data a significant correlation between the length of the replicated DNA template and the replication time (∼0.82, Spearman-Rank Correlation) is observed.
(0.01 MB PDF)

Figure S5 Filtered times mapped onto the 16 chromosomes of budding yeast. The filtered times mapped onto the locations of their corresponding DNA segments are shown. The shadings correspond to the ones used in Fig. 4. The orange line denotes the actual filtered time in seconds and the red line shows the replication profile from Raghuraman and colleagues.
(0.20 MB PDF)

## References

[1] Stinchcomb DT, Struhl K, Davis RW (1979). Isolation and characterisation of a yeast chromosomal replicator.. Nature.

[2] Zannis-Hadjopoulos M, Price GB (1998). Regulatory parameters of dna replication.. Crit Rev Eukaryot Gene Expr.

[3] Françon P, Maiorano D, Méchali M (1999). Initiation of dna replication in eukaryotes: questioning the origin.. FEBS Lett.

[4] Dutta A, Bell SP (1997). Initiation of dna replication in eukaryotic cells.. Annu Rev Cell Dev Biol.

[5] Bell SP, Dutta A (2002). Dna replication in eukaryotic cells.. Annu Rev Biochem.

[6] Weigel C, Schmidt A, Rückert B, Lurz R, Messer W (1997). Dnaa protein binding to individual dnaa boxes in the escherichia coli replication origin, oric.. EMBO J.

[7] Stillman B (2005). Origin recognition and the chromosome cycle.. FEBS Lett.

[8] Aparicio OM, Stout AM, Bell SP (1999). Differential assembly of cdc45p and dna polymerases at early and late origins of dna replication.. Proc Natl Acad Sci U S A.

[9] Zou L, Stillman B (2000). Assembly of a complex containing cdc45p, replication protein a, and mcm2p at replication origins controlled by s-phase cyclin-dependent kinases and cdc7p-dbf4p kinase.. Mol Cell Biol.

[10] Nguyen VQ, Co C, Irie K, Li JJ (2000). Clb/cdc28 kinases promote nuclear export of the replication initiator proteins mcm2-7.. Curr Biol.

[11] Francis LI, Randell JCW, Takara TJ, Uchima L, Bell SP (2009). Incorporation into the prereplicative complex activates the mcm2-7 helicase for cdc7-dbf4 phosphorylation.. Genes Dev.

[12] Takeda DY, Dutta A (2005). Dna replication and progression through s phase.. Oncogene.

[13] Nick-McElhinny SA, Gordenin DA, Stith CM, Burgers PMJ, Kunkel TA (2008). Division of labor at the eukaryotic replication fork.. Mol Cell.

[14] Okazaki R, Okazaki T, Sakabe K, Sugimoto K (1967). Mechanism of dna replication possible discontinuity of dna chain growth.. Jpn J Med Sci Biol.

[15] Ogawa T, Okazaki T (1980). Discontinuous dna replication.. Annu Rev Biochem.

[16] Kunkel TA, Burgers PM (2008). Dividing the workload at a eukaryotic replication fork.. Trends Cell Biol.

[17] Burgers PMJ (2009). Polymerase dynamics at the eukaryotic dna replication fork.. J Biol Chem.

[18] Raghuraman MK, Winzeler EA, Collingwood D, Hunt S, Wodicka L (2001). Replication dynamics of the yeast genome.. Science.

[19] Wintersberger E (2000). Why is there late replication?. Chromosoma.

[20] Zhang Z, Shibahara K, Stillman B (2000). Pcna connects dna replication to epigenetic inheritance in yeast.. Nature.

[21] Ji N, Allshire RC, Klar AJ, Grewal SI (2001). A role for dna polymerase alpha in epigenetic control of transcriptional silencing in fission yeast.. EMBO J.

[22] Mechali M (2001). Dna replication origins: from sequence specificity to epigenetics.. Nat Rev Genet.

[23] Pasero P, Bensimon A, Schwob E (2002). Single-molecule analysis reveals clustering and epigenetic regulation of replication origins at the yeast rdna locus.. Genes Dev.

[24] Antequera F (2004). Genomic specification and epigenetic regulation of eukaryotic dna replication origins.. EMBO J.

[25] Zhou J, Chau C, Deng Z, Stedman W, Lieberman PM (2005). Epigenetic control of replication origins.. Cell Cycle.

[26] Hamlin JL, Mesner LD, Lar O, Torres R, Chodaparambil SV (2008). A revisionist replicon model for higher eukaryotic genomes.. J Cell Biochem.

[27] Tabancay AP, Forsburg SL (2006). Eukaryotic dna replication in a chromatin context.. Curr Top Dev Biol.

[28] Kohzaki H, Ito Y, Murakami Y (1999). Context-dependent modulation of replication activity of saccharomyces cerevisiae autonomously replicating sequences by transcription factors.. Mol Cell Biol.

[29] Nieduszynski CA, Blow JJ, Donaldson AD (2005). The requirement of yeast replication origins for pre-replication complex proteins is modulated by transcription.. Nucleic Acids Res.

[30] Mori S, Shirahige K (2007). Perturbation of the activity of replication origin by meiosis-specific transcription.. J Biol Chem.

[31] Lucchini R, Sogo JM (1994). Chromatin structure and transcriptional activity around the replication forks arrested at the 3′ end of the yeast rrna genes.. Mol Cell Biol.

[32] Deshpande AM, Newlon CS (1996). Dna replication fork pause sites dependent on transcription.. Science.

[33] Wellinger RE, Prado F, Aguilera A (2006). Replication fork progression is impaired by transcription in hyperrecombinant yeast cells lacking a functional tho complex.. Mol Cell Biol.

[34] Chabes A, Stillman B (2007). Constitutively high dntp concentration inhibits cell cycle progression and the dna damage checkpoint in yeast saccharomyces cerevisiae.. Proc Natl Acad Sci U S A.

[35] Pruitt KD, Tatusova T, Maglott DR (2007). Ncbi reference sequences (refseq): a curated non-redundant sequence database of genomes, transcripts and proteins.. Nucleic Acids Res.

[36] Kirkpatrick S, Gelatt CD, Vecchi MP (1983). Optimization by simulated annealing.. Science.

[37] Zhu C, Byrd RH, Lu P, Nocedal J (1997). Algorithm 778: L-bfgs-b: Fortran subroutines for large-scale bound-constrained optimization.. ACM Trans Math Softw.

[38] Spearman C (1987). The proof and measurement of association between two things.. The American Journal of Psychology.

[39] Holm S (1979). A simple sequentially rejective multiple test procedure.. Scand J Stat.

[40] Akaike H (1974). A new look at the statistical model identification.. IEEE Trans Autom.

[41] R-Development-Core-Team (2008). R: A language and environment for statistical computing. R Foundation for Statistical Computing, Vienna, Austria..

[42] Spiesser TW, Klipp E, Barberis M (2009). A model for the spatiotemporal organization of dna replication in saccharomyces cerevisiae.. Mol Genet Genomics.

[43] Donaldson AD, Raghuraman MK, Friedman KL, Cross FR, Brewer BJ (1998). Clb5-dependent activation of late replication origins in s. cerevisiae.. Mol Cell.

[44] McCune HJ, Danielson LS, Alvino GM, Collingwood D, Delrow JJ (2008). The temporal program of chromosome replication: genome-wide replication in clb5Delta saccharomyces cerevisiae.. Genetics.

[45] Hiraga SI, Hagihara-Hayashi A, Ohya T, Sugino A (2005). Dna polymerases alpha, delta, and epsilon localize and function together at replication forks in saccharomyces cerevisiae.. Genes Cells.

