# Supplementary figures and images for: What Influences DNA Replication Rate in Budding Yeast?
Source: PLoS One. 2010 Apr 27;5(4):e10203. doi: 10.1371/journal.pone.0010203 (PMC2860512; doi:10.1371/journal.pone.0010203)

Figure S1

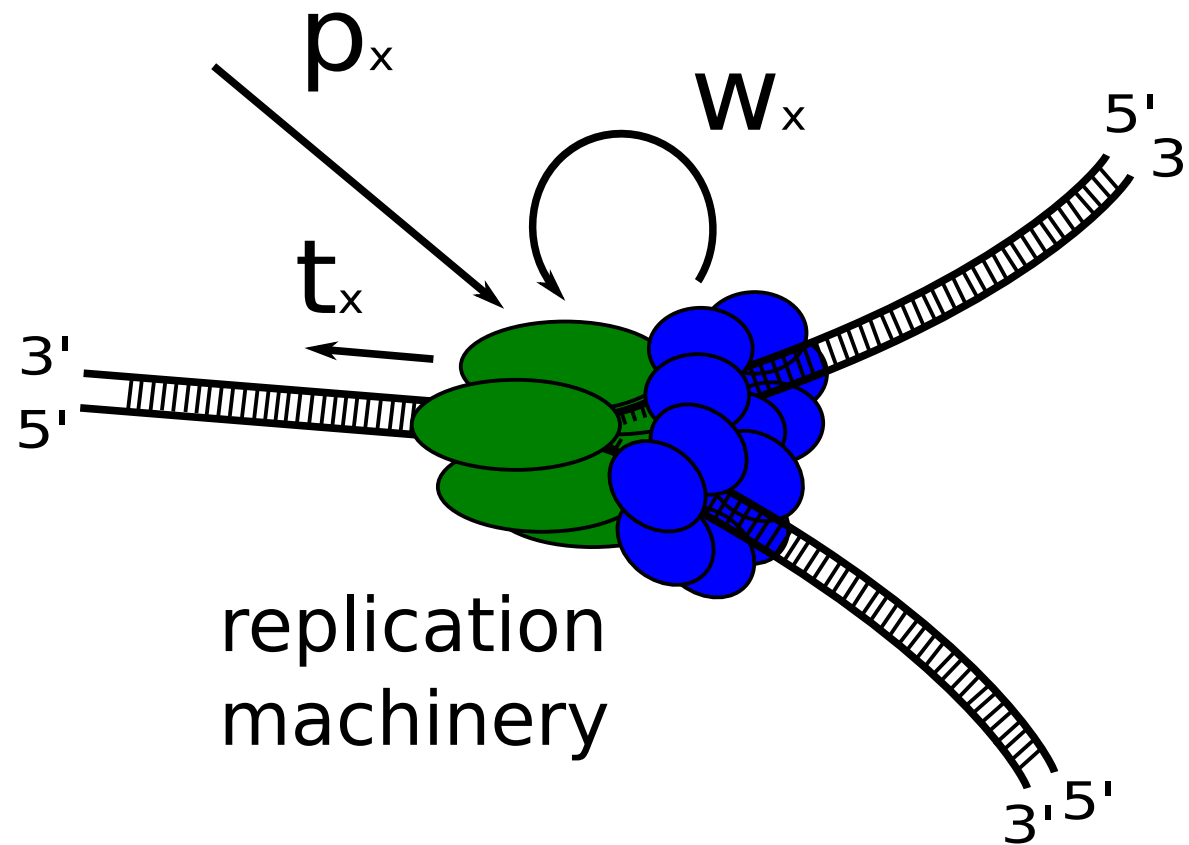

Supplement: Figure S1 — Schematic view of the DNA replication model. The replication machinery can move forward with a base-dependent probability p(X) for base X, taking a mean time t(X) for the forward step and a mean time w(X) for the waiting step. (0.03 MB PDF) [file pone.0010203.s001.pdf]

Figure S2

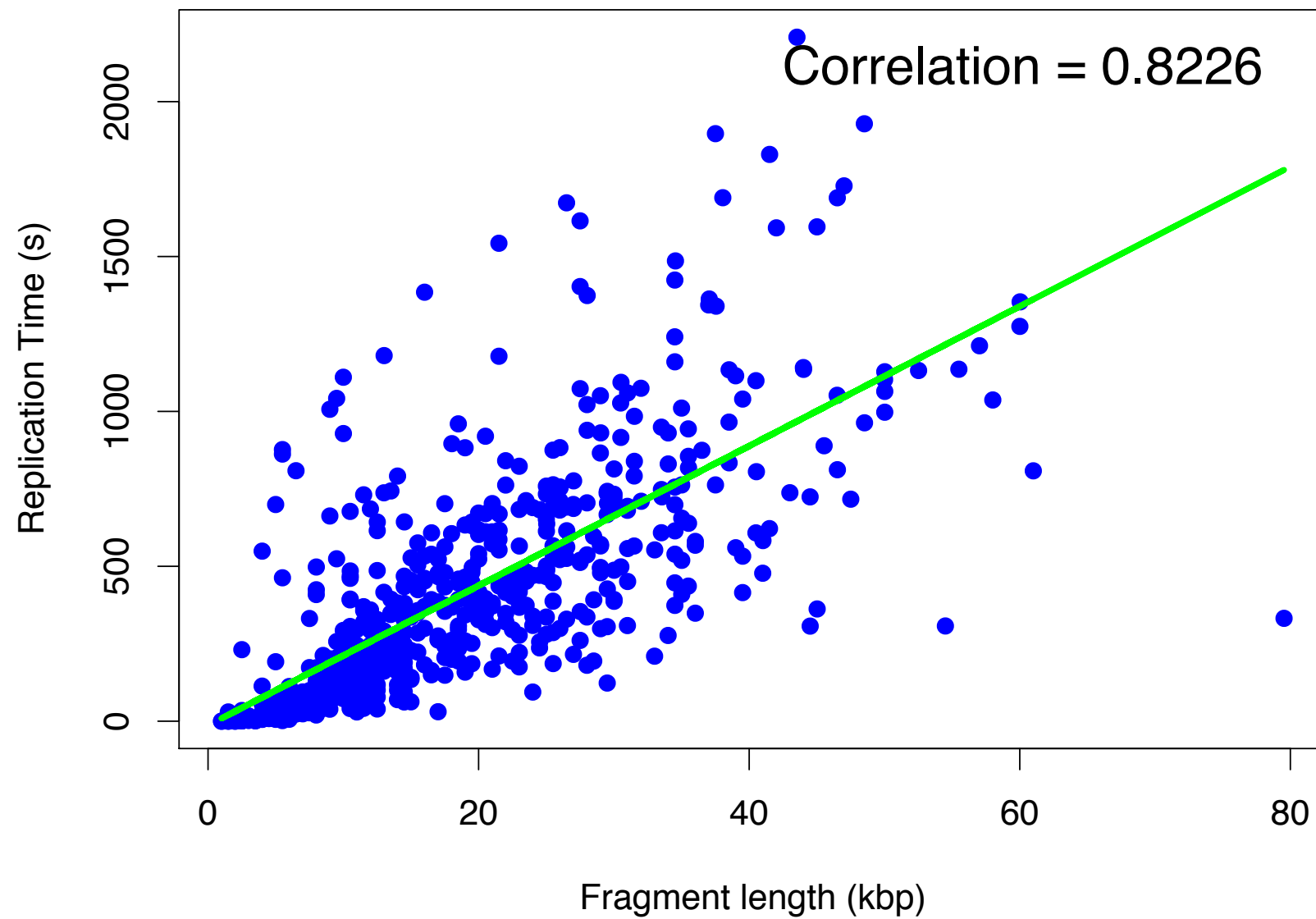

Supplement: Figure S2 — Estimated parameters for model 1. Histograms for the 12 parameters as obtained from 1000 independent optimization runs with uniformly distributed initial values. CV denotes coefficient of variation (standard deviation/mean). (0.04 MB PDF) [file pone.0010203.s002.pdf]

Figure S3

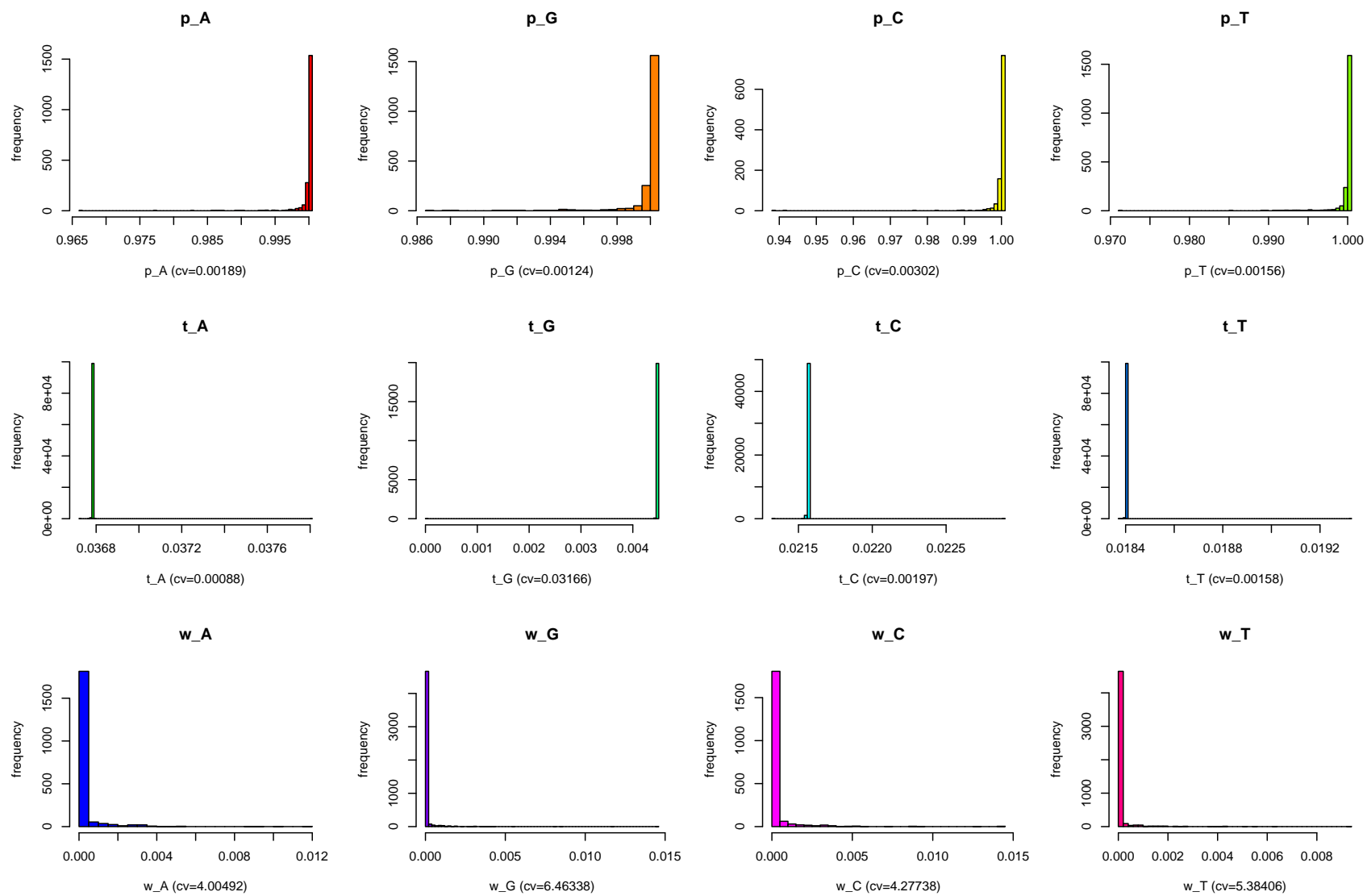

Supplement: Figure S3 — Estimated parameters for model 2. Histograms for the 3 parameters as obtained from 1000 independent optimization runs with uniformly distributed initial values. CV denotes coefficient of variation (standard deviation/mean). (0.02 MB PDF) [file pone.0010203.s003.pdf]

Figure S4

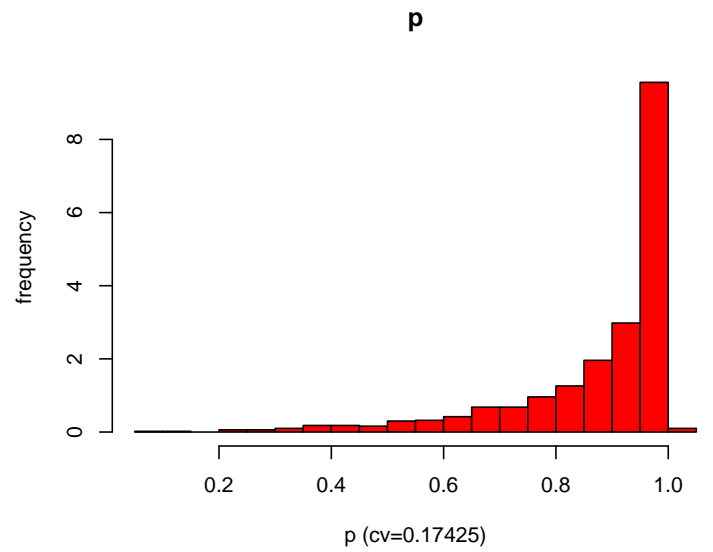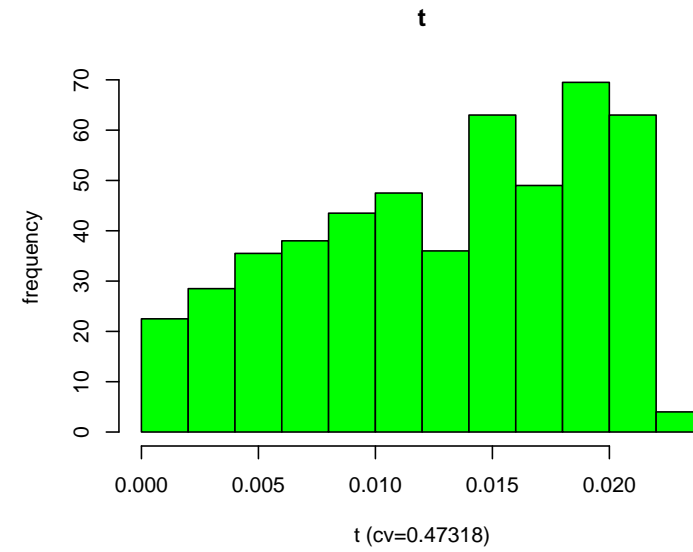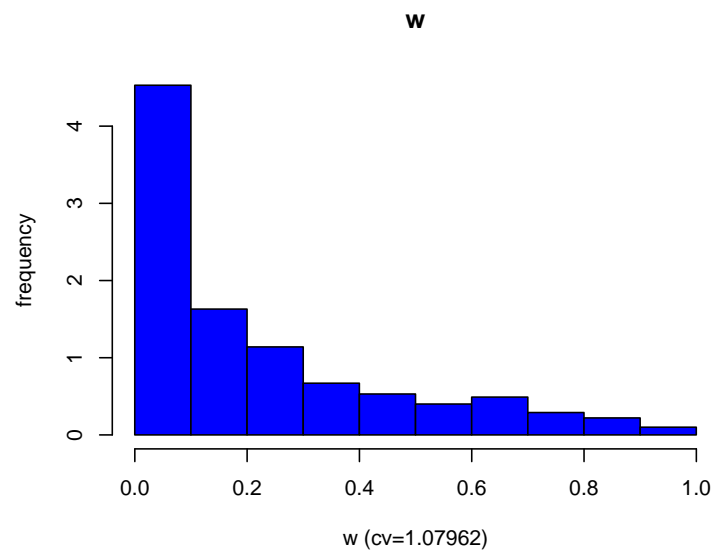

Supplement: Figure S4 — Dependence of replication times on the lengths of the DNA templates. In the experimental data a significant correlation between the length of the replicated DNA template and the replication time (∼0.82, Spearman-Rank Correlation) is observed. (0.01 MB PDF) [file pone.0010203.s004.pdf]

Figure S5-A

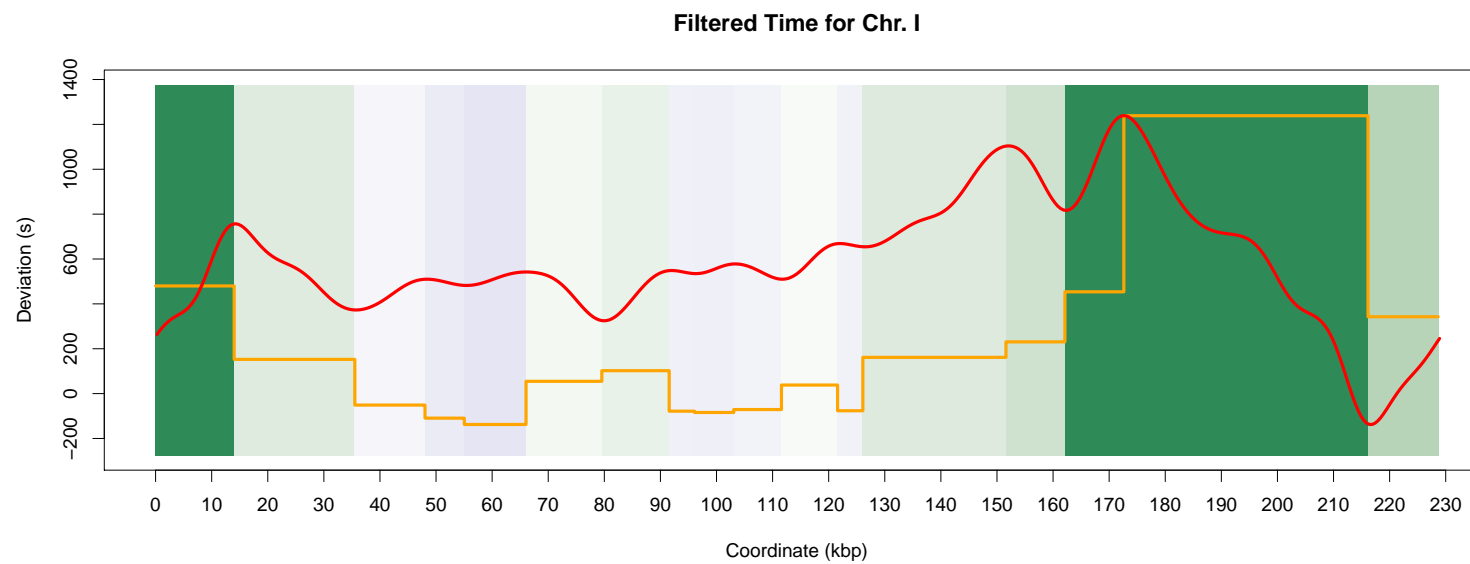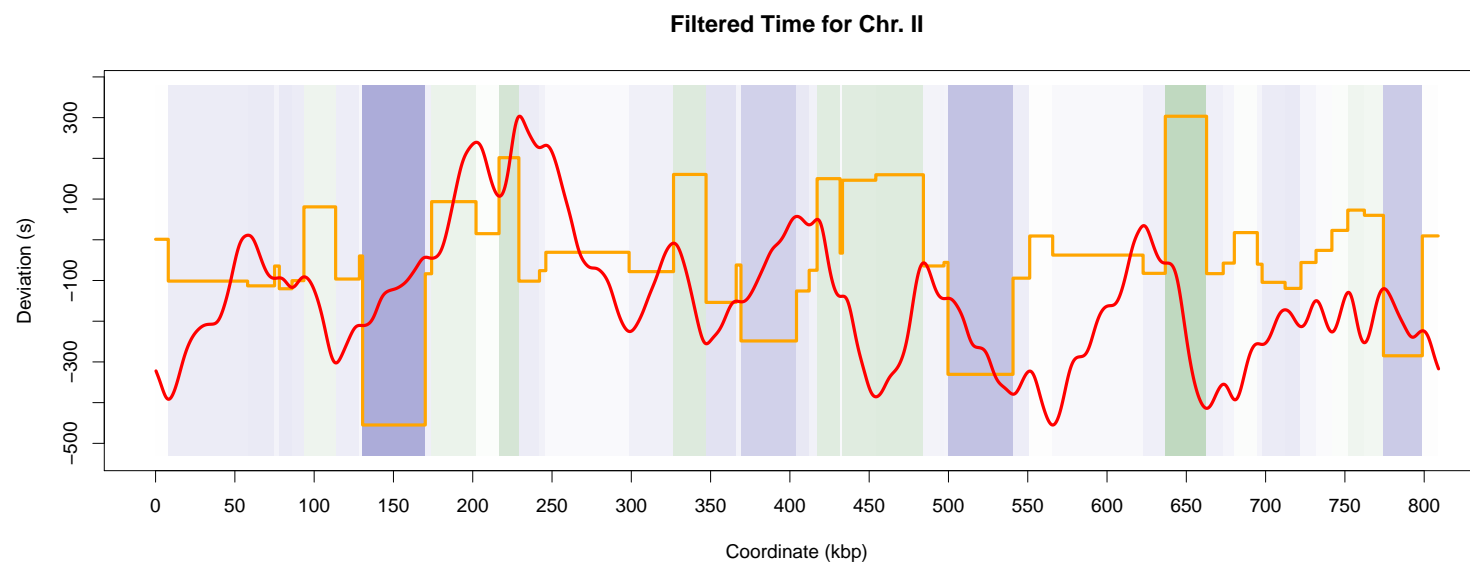

Figure S5-B

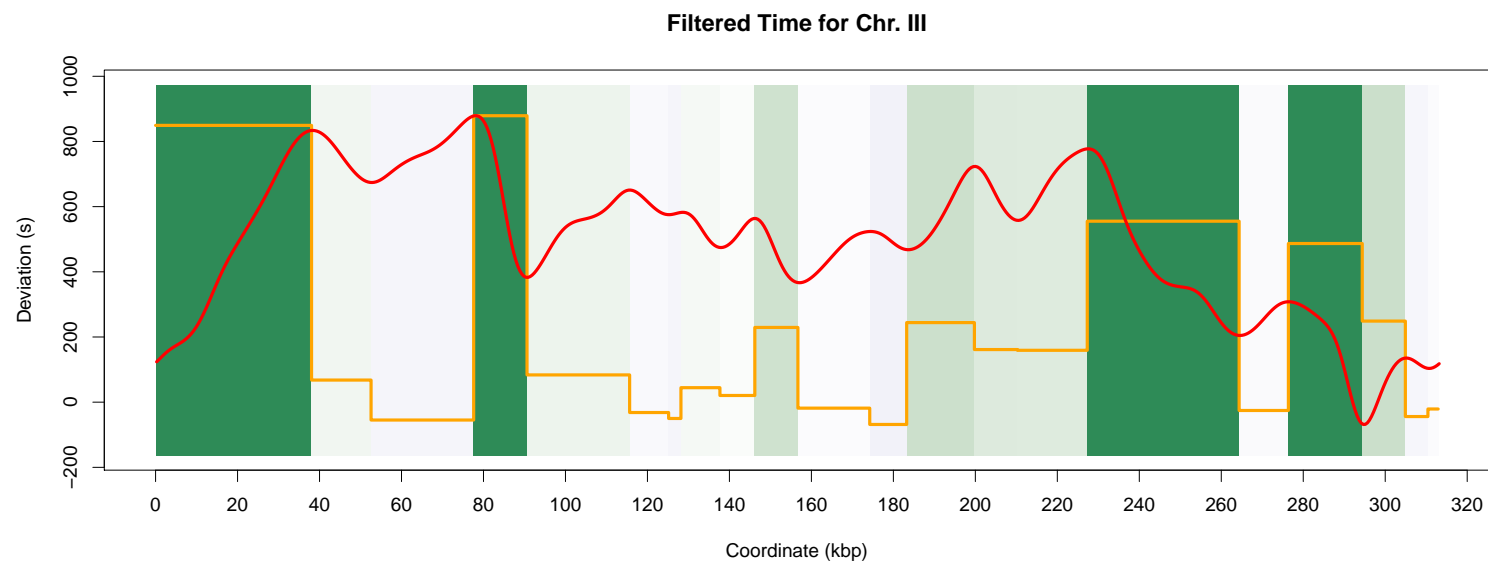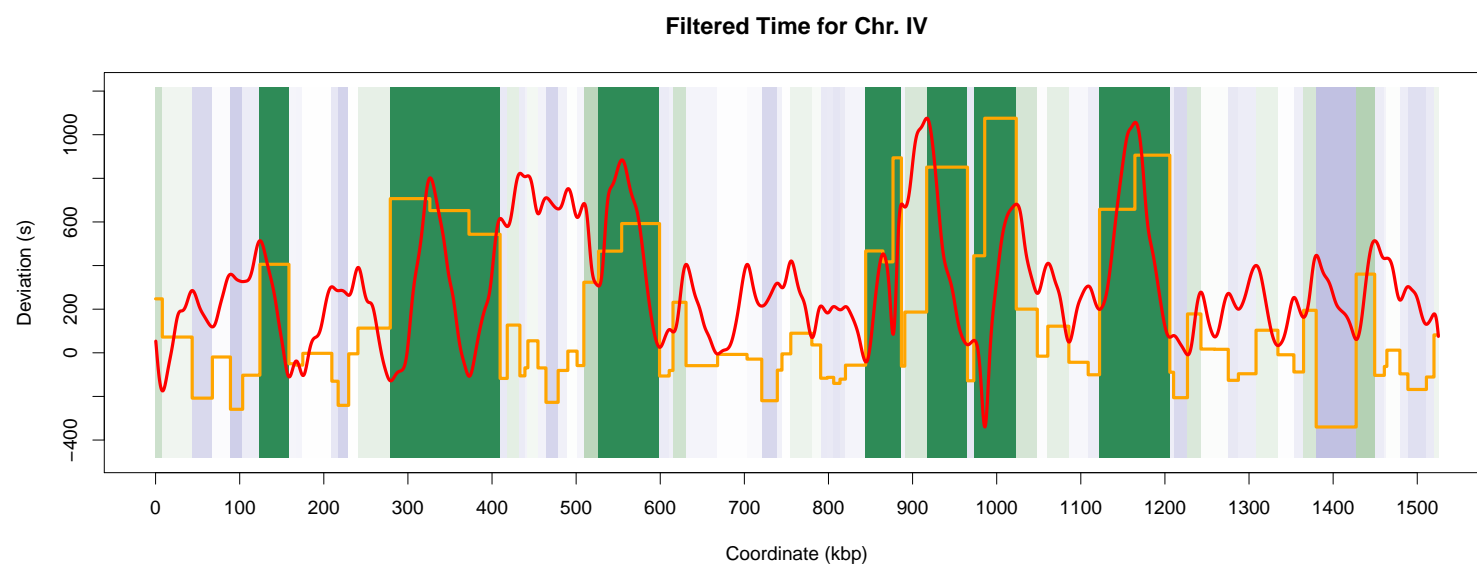

Figure S5-C

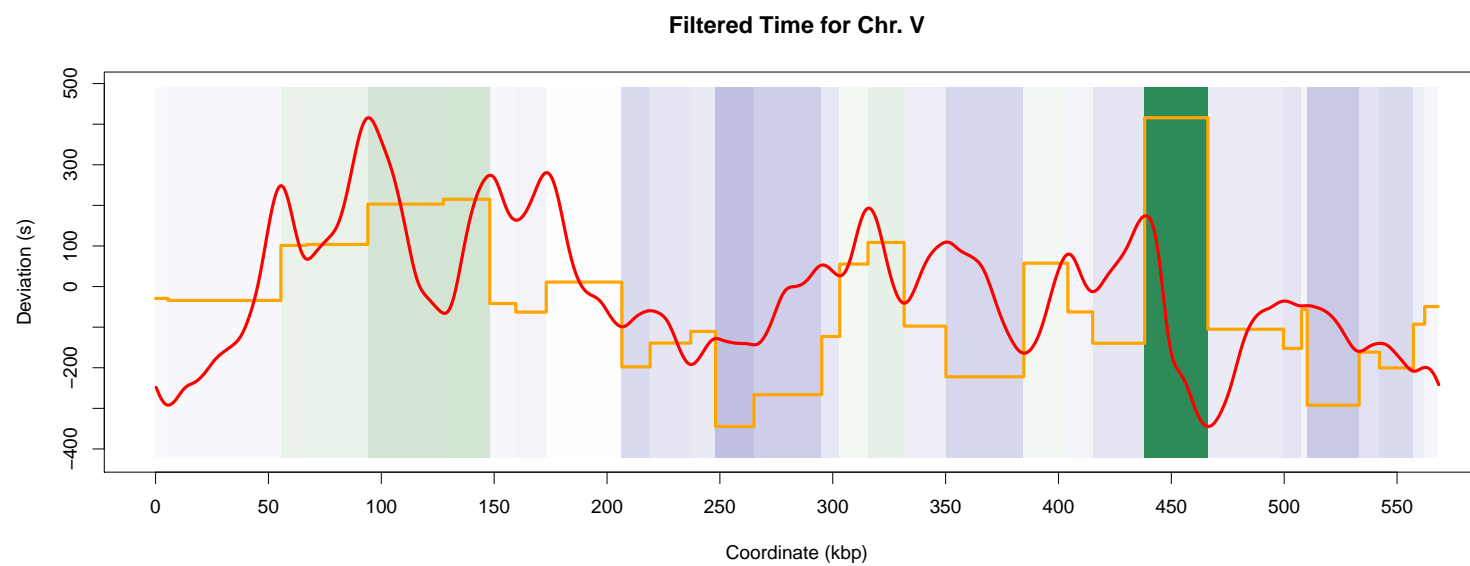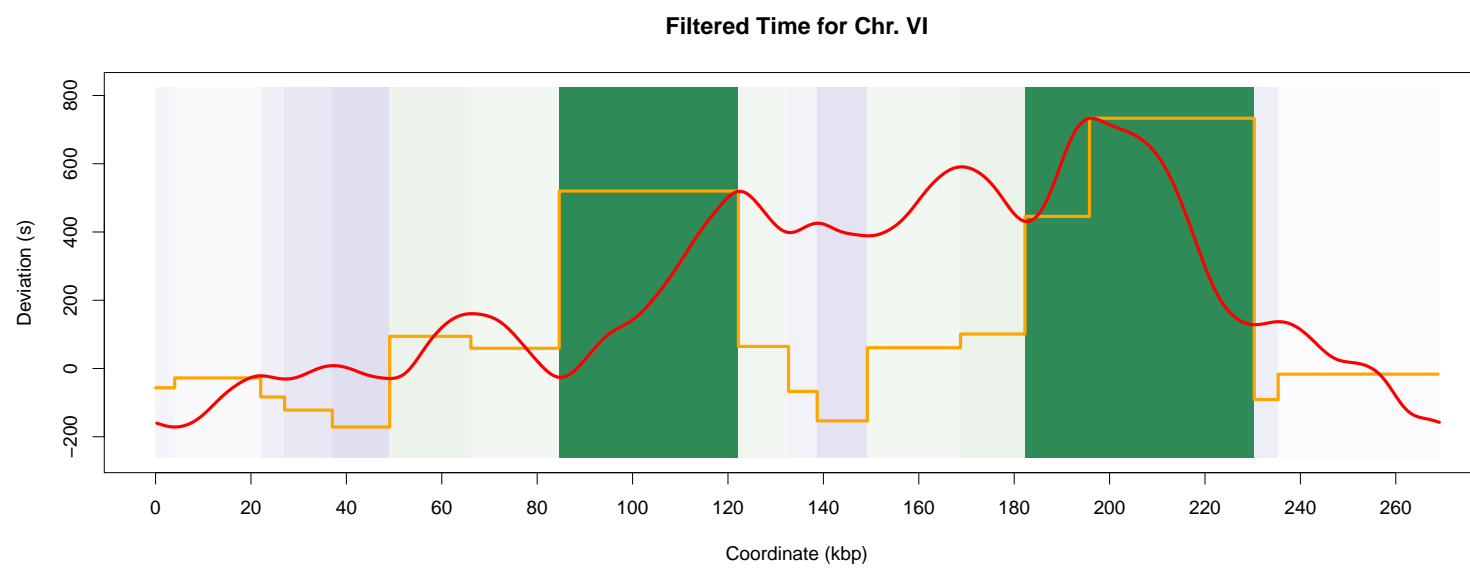

Figure S5-D

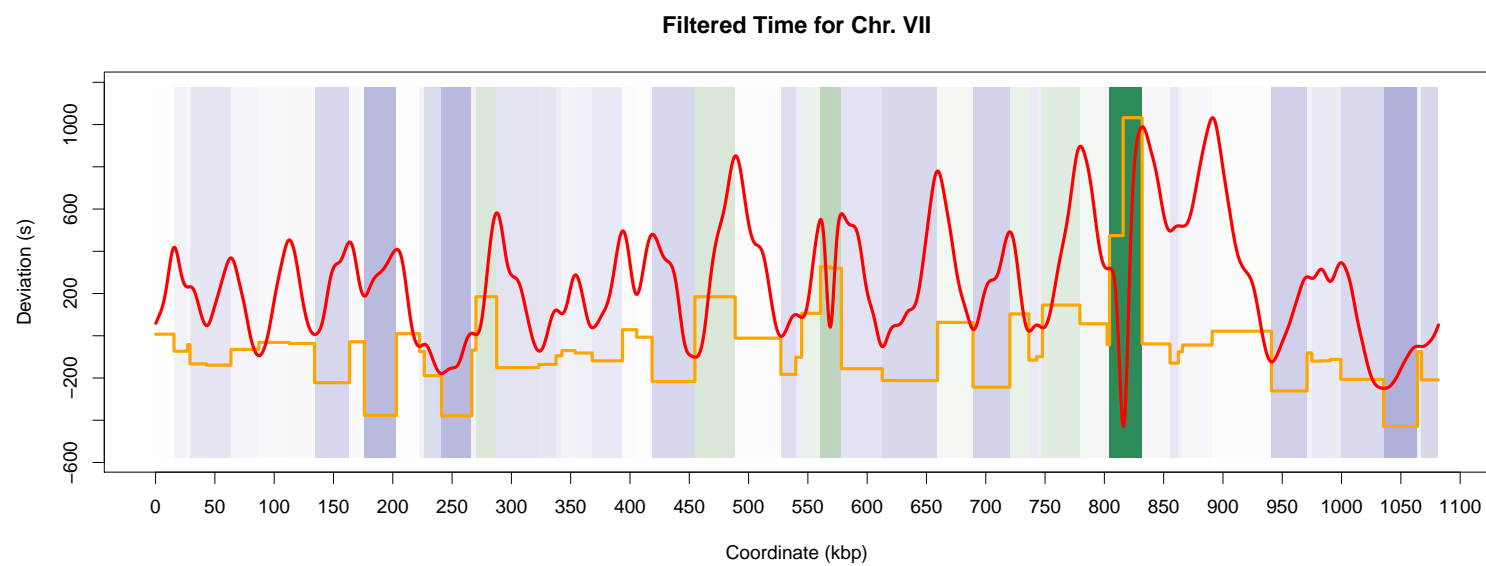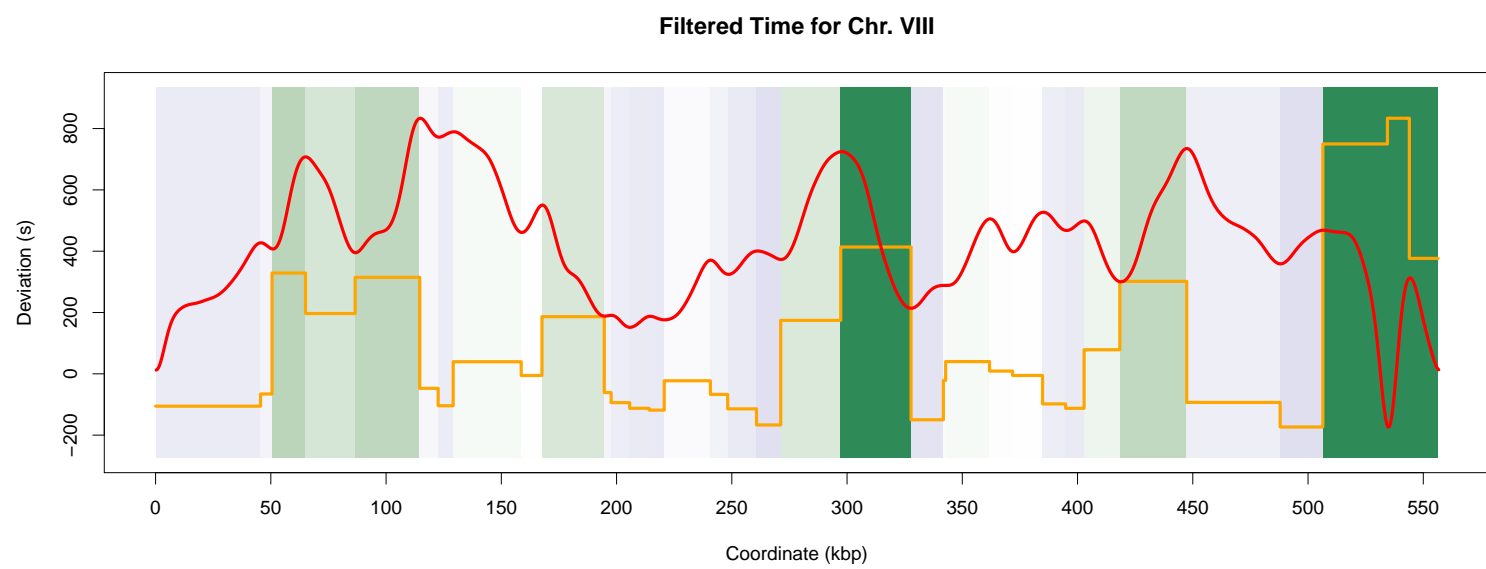

Figure S5-E

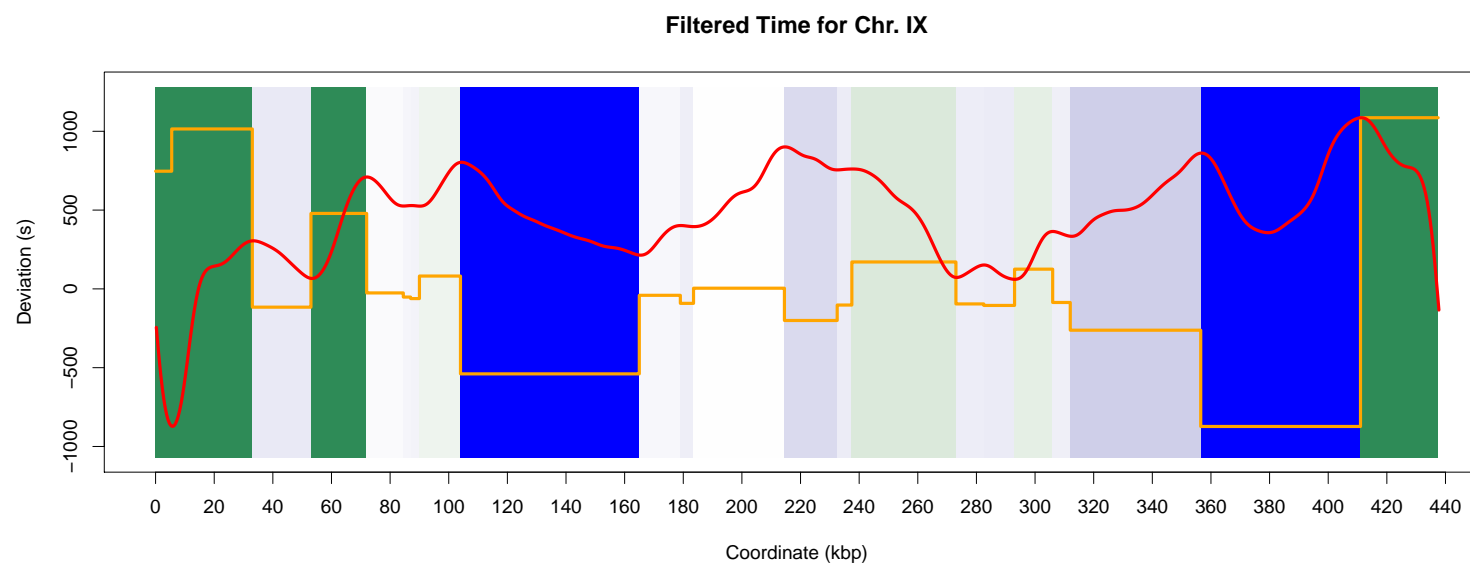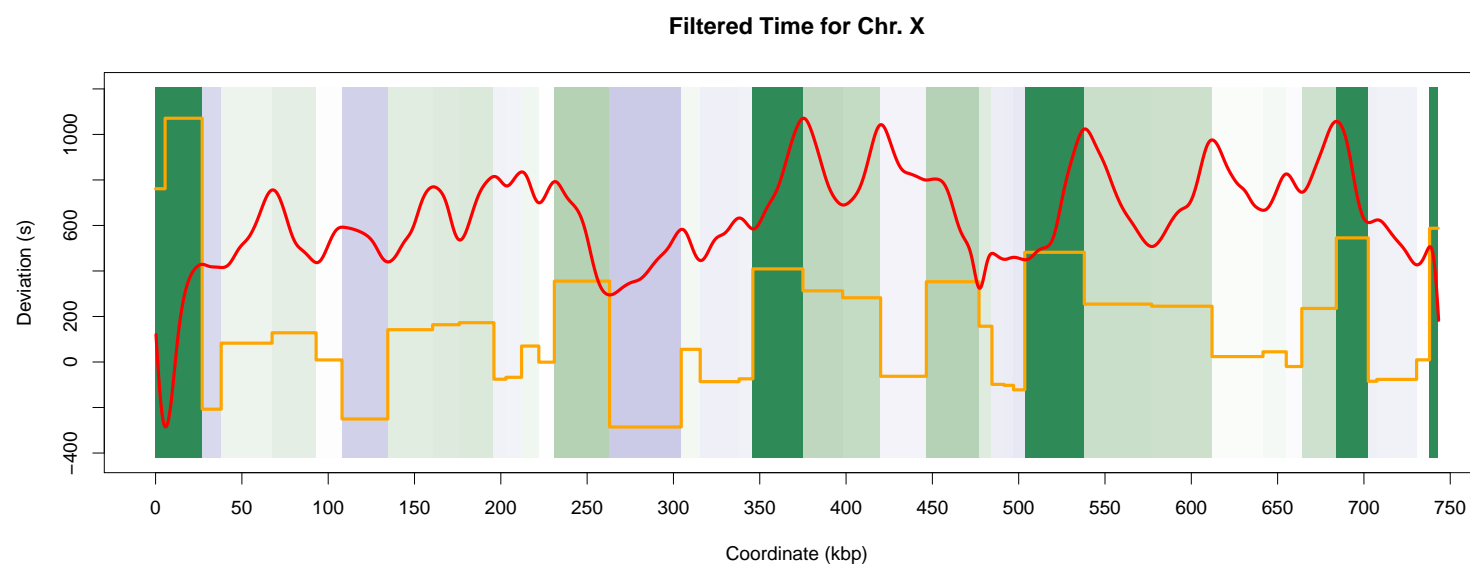

Figure S5-F

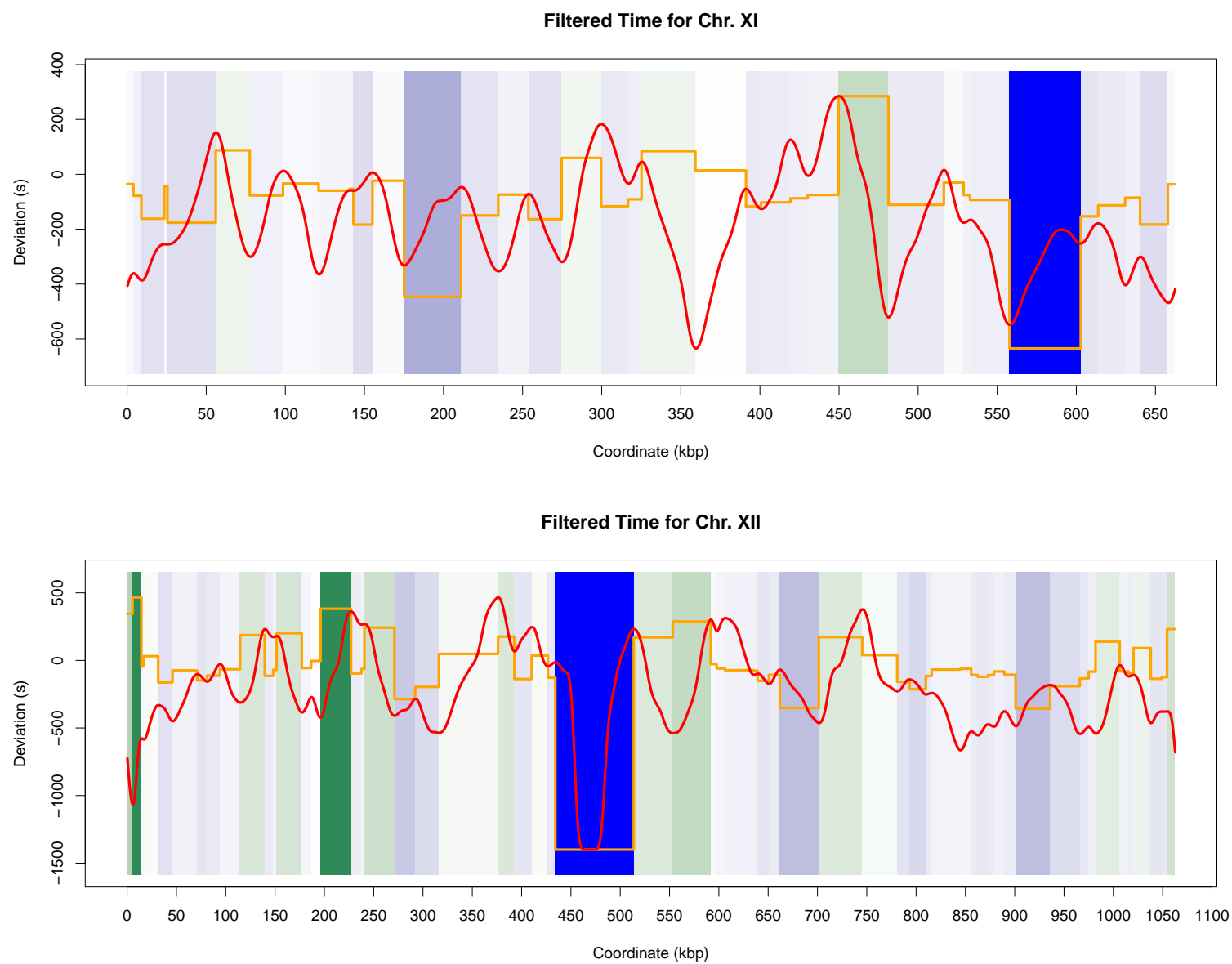

Figure S5-G

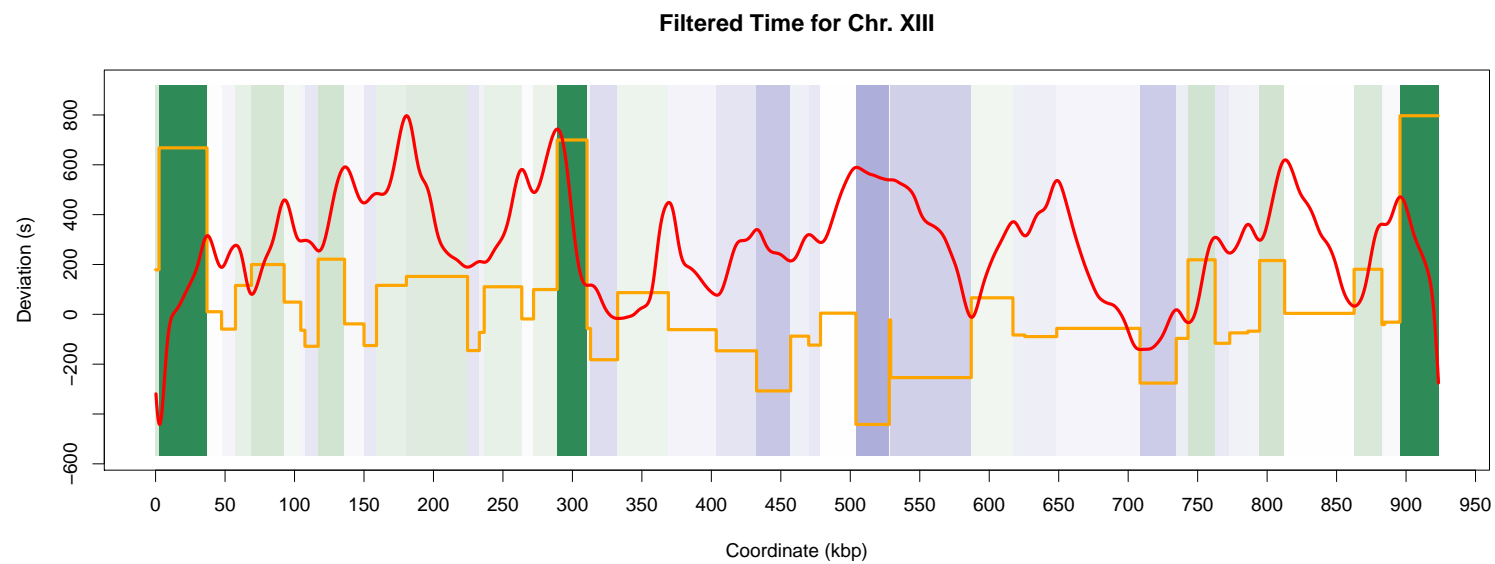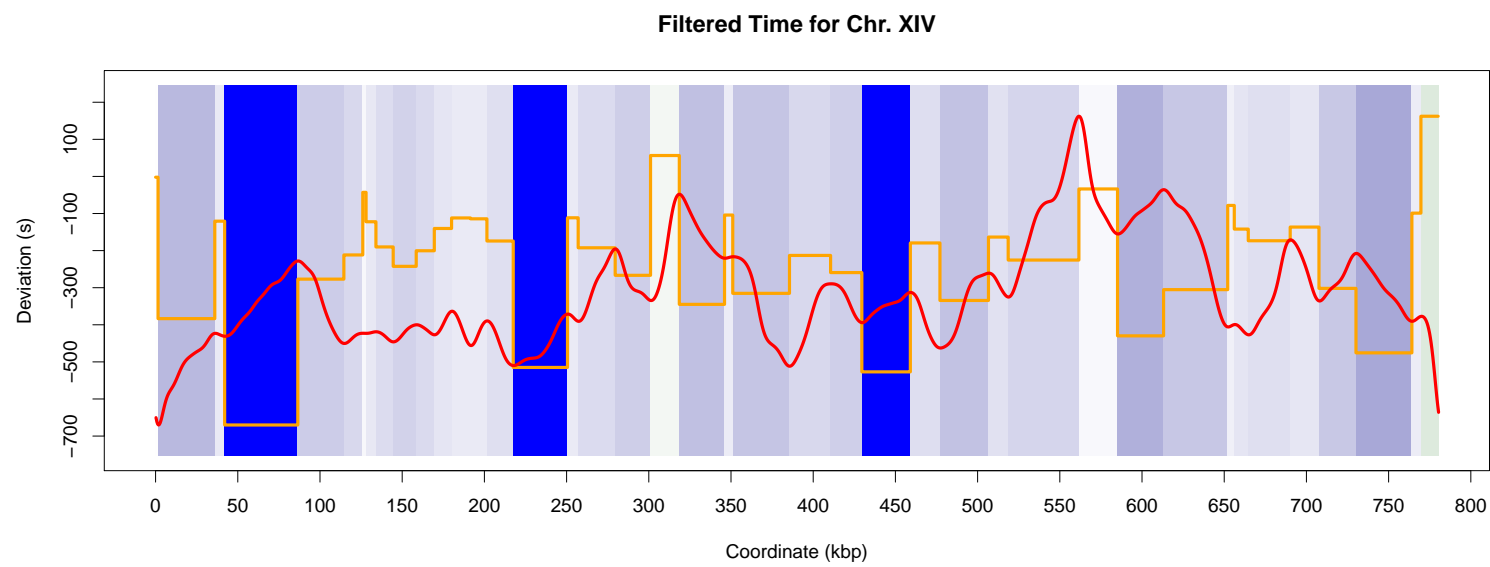

Figure S5-H

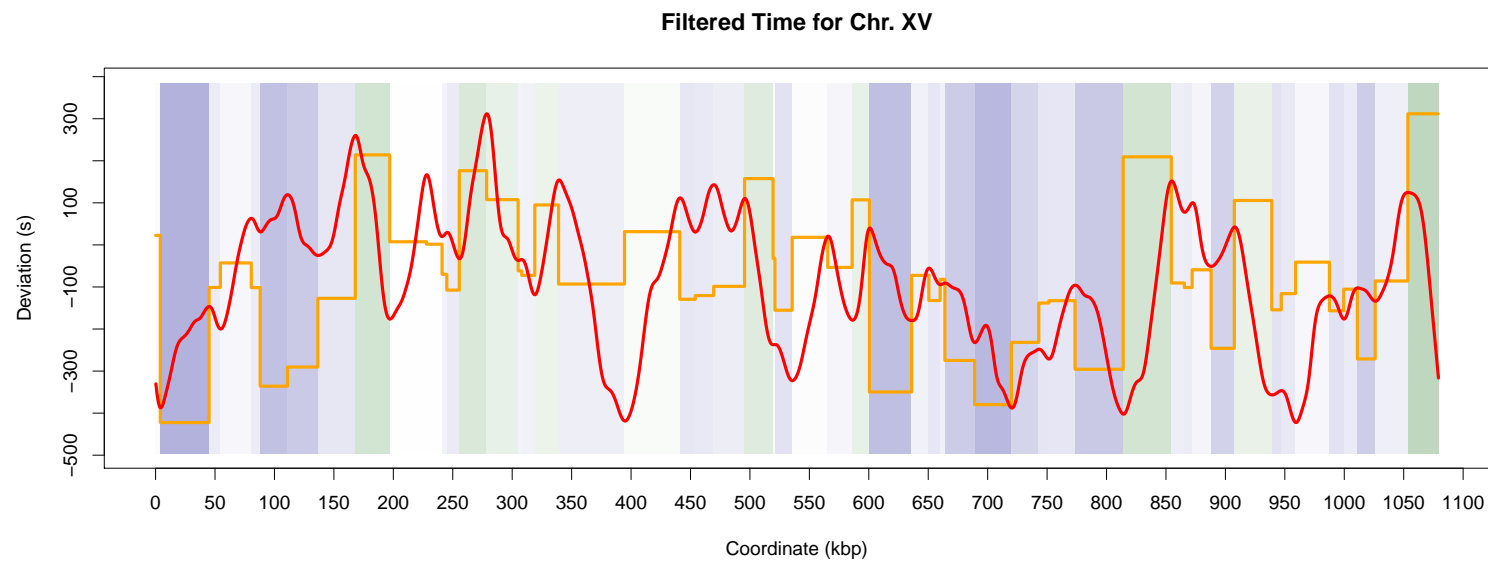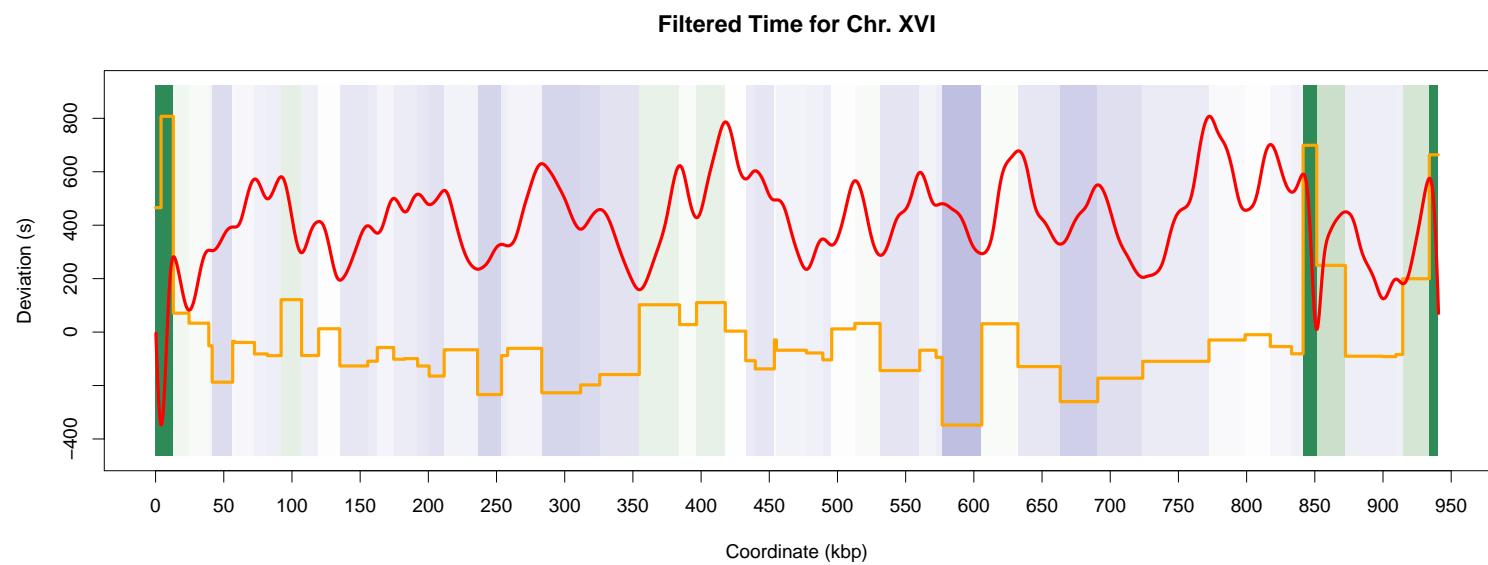

Supplement: Figure S5 — Filtered times mapped onto the 16 chromosomes of budding yeast. The filtered times mapped onto the locations of their corresponding DNA segments are shown. The shadings correspond to the ones used in Fig. 4. The orange line denotes the actual filtered time in seconds and the red line shows the replication profile from Raghuraman and colleagues. (0.20 MB PDF) [file pone.0010203.s005.pdf]
